# Multi-omics analysis reveals structural and transcriptional regulation specificity underlying differential benzylisoquinoline alkaloid accumulation in *Coptis*

**DOI:** 10.1093/hr/uhaf338

**Published:** 2025-12-15

**Authors:** Xufang Tian, Siyu Yang, Siyu Wang, Wei Li, Guofeng Li, Shi Zhang, Jin Wang, Di Liu, Yifei Liu

**Affiliations:** College of Pharmacy, Hubei University of Chinese Medicine, Wuhan, Hubei 430065, China; College of Pharmacy, Hubei University of Chinese Medicine, Wuhan, Hubei 430065, China; College of Pharmacy, Hubei University of Chinese Medicine, Wuhan, Hubei 430065, China; College of Pharmacy, Hubei University of Chinese Medicine, Wuhan, Hubei 430065, China; College of Pharmacy, Hubei University of Chinese Medicine, Wuhan, Hubei 430065, China; College of Pharmacy, Hubei University of Chinese Medicine, Wuhan, Hubei 430065, China; College of Pharmacy, Hubei University of Chinese Medicine, Wuhan, Hubei 430065, China; College of Pharmacy, Hubei University of Chinese Medicine, Wuhan, Hubei 430065, China; Hubei Shizhen Laboratory, Wuhan, Hubei 430060, China; College of Pharmacy, Hubei University of Chinese Medicine, Wuhan, Hubei 430065, China; Hubei Shizhen Laboratory, Wuhan, Hubei 430060, China

## Abstract

*Coptis* species are rich in protoberberine-type benzylisoquinoline alkaloids (BIAs). However, the differential BIA accumulation between *Coptis chinensis* and *C. teeta*, two primary botanical sources of traditional Chinese medicine ‘Huanglian’, remains mechanistically poorly understood. Here, we combined widely targeted metabolomics, matrix-assisted laser desorption/ionization mass spectrometry imaging, histological characterization, and transcriptomic analyses to investigate the mechanisms underlying the specialized BIA accumulation in *C. chinensis* versus *C. teeta*. Clearly, we observed significantly elevated BIA accumulation in *C. chinensis* rhizomes compared to *C. teeta*, in particular, the preferential BIA localization within the cortical tissues of *C. chinensis* rhizomes, consistent with the anatomically expanded cortical and xylem regions. This structural specialization facilitates BIA compartmental distribution patterns. Integrated transcriptomic–metabolomic analysis further constructed a BIA biosynthetic regulatory network, identifying key transcription factors that synergistically promote BIA accumulation in *C. chinensis* rhizomes, establishing their roles as speciation-associated regulators of medicinal quality divergence between *C. chinensis* and *C. teeta*. Overall, this study provides the first integrated anatomical and transcriptional framework explaining interspecies differences in BIA accumulation, enabling the development of quality improvement strategies for medicinal plants.

## Introduction

Traditional Chinese medicine (TCM) has served as a foundation of human health for thousands of years. The therapeutic efficacy of medicinal herbs derives from a diverse array of active constituents, including alkaloids [1, 2], polysaccharides [3, 4], flavonoids [5], and terpenoids [6, 7], which constitute the material basis of their pharmacological actions. Among these, the goldthread genus *Coptis* is notably rich in protoberberine-type benzylisoquinoline alkaloids (BIAs) [8]. More than 20 BIAs have been recognized in *Coptis*, making up 5%–10% of the total metabolites [9]. The dried rhizomes of *Coptis* species, known as Coptidis Rhizoma (CR) in TCM, have been used therapeutically for over 2000 years [8]. The diverse BIAs serve as both the primary bitter compounds and bioactive constituents of CR [10–12]. Three species, *Coptis chinensis*, *C. teeta*, and *C. deltoidea*, are recognized as the standard botanical sources of CR in the Chinese Pharmacopoeia. However, accumulating evidence revealed interspecific variations in BIA content among them [12–16]. For instance, comparative quality assessments of CR indicate that *C. chinensis* is the optimal source for columbamine, coptisine, and palmatine, whereas *C. teeta* contains the highest berberine levels, along with moderately elevated jatrorrhizine and magnoflorine [14–16]. Notably, *C. chinensis* uniquely accumulates epiberberine, which is less detected in *C. teeta* [14–17]. Despite these observed differences, the mechanisms underlying interspecific BIA variation remain unclear.

The variation of plant natural products across species and tissues is a complex biological phenomenon, driven by organ specificity, genotypic variation, and environmental influences that collectively shape secondary metabolite biosynthesis and accumulation [18]. Notably, the compartmentalized synthesis of secondary metabolites often correlates with specialized tissue anatomical structures. In *Papaver somniferum*, morphine alkaloids are exclusively synthesized and stored in laticifers, with immature capsules exhibiting significantly higher concentrations due to their dense laticifer networks [19, 20]. Spatial distribution mapping in *Panax ginseng* reveals the predominant accumulation of ginsenosides in the root cork layer, stem epidermis, and leaf adaxial epidermis. A 2.3-fold increase in the production of the rare ginsenoside Rg3 was achieved by genetically modulating the epidermal patterning factor (PgEPF) and a TCP family transcription factor (PgTCP) to thicken the leaf epidermis [21]. An analogous tissue-specific accumulation pattern occurs in *Taxus* species, where taxol and its biosynthetic intermediates concentrate selectively in the endodermis of young stems [22].

Genetic factors also constitute the core regulatory framework for metabolic divergence between species. Differential expression of key pathway genes as well as transcription factors (TFs) directly modulates compound-specific synthesis rates and accumulation levels [18]. In *Salvia miltiorrhiza*, ecotype-specific phenolic acid biosynthesis arises from a C/G polymorphism at position 146 of a *rosmarinic acid synthase (RAS)* gene [18]. This mutation alters substrate affinity in the RAS enzyme, redirecting metabolic flux toward either rosmarinic acid or caffeoylquinic acid. Further divergence is driven by the ecotype-restricted TF *SmWRKY40*, which coordinately upregulates rosmarinic acid, salvianolic acid B, and lignin biosynthesis [18]. Similarly, domesticated tartary buckwheat (*Fagopyrum tataricum*) populations exhibit a metabolic trade-off relative to wild counterparts, with rutin, a therapeutically vital flavonoid, declining by 30%–50%. Genome-wide association studies implicate 588 selection-enriched loci within flavonoid biosynthetic pathways, systemically downregulating *chalcone synthase (CHS)* and attenuating secondary metabolite production in cultivated accessions [23]. Comparatively, the pattern remains unestablished for how specific gene regulatory networks drive BIA divergence across *Coptis* species and tissues.

The biosynthesis of major BIAs in *Coptis* involves three sequential stages: common precursor formation, intermediate biosynthesis, and end BIA product assembly [24]. Initially, dopamine and 4-hydroxyphenylacetaldehyde are converted into the central precursor (*S*)-reticuline via sequential enzymatic reactions catalyzed by norcoclaurine synthase (NCS), 6-*O*-methyltransferase (6OMT), coclaurine *N*-methyltransferase (CNMT), (*S*)-*N*-methylcoclaurine 3′-hydroxylase (NMCH/CYP80B1), and 4′-*O*-methyltransferase (4′OMT) [25–27]. Subsequently, (*S*)-reticuline serves as the substrate for divergent metabolic pathways catalyzed by enzymes including berberine bridge enzyme (BBE), cytochrome P450s (CYP80G2, CYP719), and methyltransferases (SOMT, 6DM, OMT1), generating nine BIA intermediates [24, 28–31]. Finally, the terminal stage yields five principal protoberberine alkaloids (columbamine, demethyleneberberine, berberine, coptisine, epiberberine), likely mediated by *S*-tetrahydroprotoberberine oxidase (STOX) [32]. Columbamine and demethyleneberberine are further methylated by an *O*-methyltransferase (OMT) to form palmatine and jatrorrhizine, respectively, while magnoflorine biosynthesis proceeds via an alternative pathway involving the enzymatic conversion of (*S*)-corytuberine by reticuline *N*-methyltransferase (RNMT) or CNMT [24, 25, 27, 30]. TFs serve as master regulators that orchestrate the expression of pathway genes. Functional studies in *Coptis* have characterized key TFs regulating BIA biosynthesis, including bHLHs (CcbHLH001, CcbHLH002, CjbHLH2) and WRKY-type regulators (CcWRKY7, CcWRKY29, CcWRKY32) [33–35]. Nevertheless, current understanding of TF-mediated regulatory networks remains fragmented, particularly concerning species-specific regulators in *Coptis*.

Appearing metabolomic methods, such as matrix-assisted laser desorption/ionization mass spectrometry imaging (MALDI-MSI), which integrates mass spectrometry and imaging technologies, allow for high-resolution capture of spatial distribution information of plant metabolites at the tissue and cellular level, making it a highly favored molecular imaging tool [36]. In this study, we employed widely targeted metabolomics and MALDI-MSI to characterize the differential BIA accumulation patterns between *C. chinensis* and *C. teeta* in rhizome and fibrous root tissues. We identified preferential BIA biosynthesis and accumulation within the rhizome cortex of *C. chinensis*. Subsequent histological analysis demonstrated marked cortical thickening in *C. chinensis* rhizomes compared to *C. teeta*, suggesting structural adaptation underpinning BIA accumulation. Through integrated multi-omics analysis, we further elucidated a rhizome-specific transcriptional network governed by GRAS, bHLH, HB, MYB, C3H, and SBP TFs, contributing to the high BIA accumulation in *C. chinensis* rhizomes.

## Results

### Divergent accumulation of BIAs between *Coptis* species and tissues

**Figure 1:**
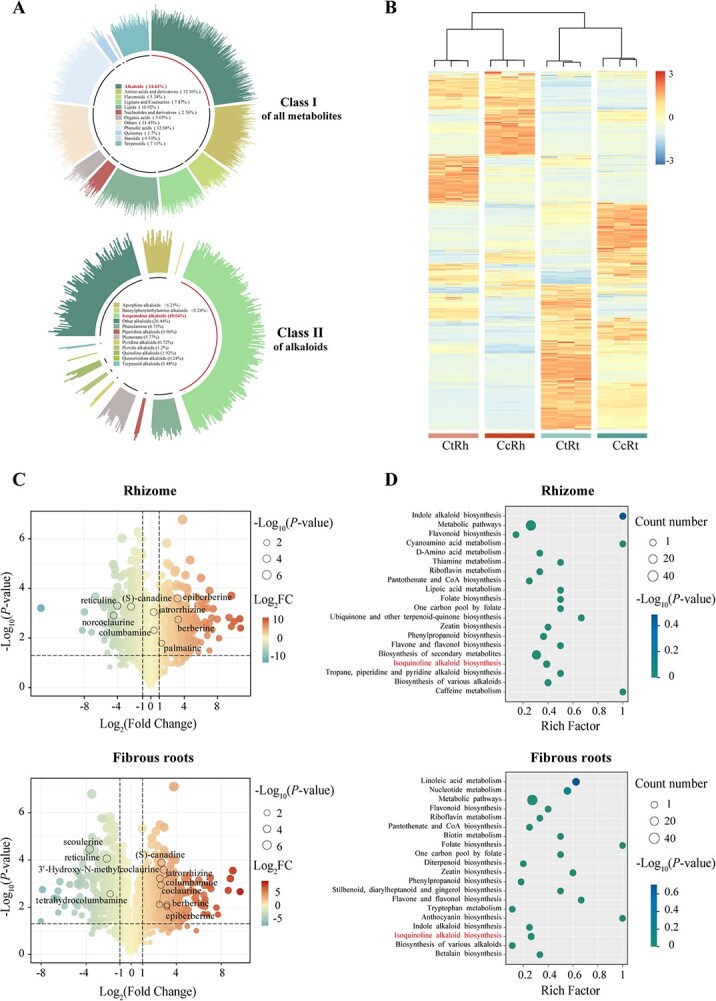
Widely targeted metabolomics comparative analysis of *C. chinensis* and *C. teeta*. (A) Primary classification of metabolites detected in *Coptis*, and secondary classification of alkaloids. (B) Heatmap of metabolites identified across *C. chinensis* rhizome (CcRh), *C. teeta* rhizome (CtRh), *C. chinensis* fibrous root (CcRt), and *C. teeta* fibrous root (CtRt) samples. (C) Volcano plots of differential metabolites between *C. chinensis* and *C. teeta* rhizomes/fibrous roots. (D) KEGG enrichment analysis of upregulated differential metabolites in *C. chinensis* compared to *C. teeta* rhizomes/fibrous roots.

The rhizomes and fibrous roots of *Coptis* species serve as the primary medicinal materials in clinical practice due to their high concentration of bioactive compounds. To investigate species- and tissue-specific accumulation patterns of specialized metabolites, we performed widely targeted metabolomics profiling on rhizomes and fibrous roots of *C. chinensis* and *C. teeta* using ultra performance liquid chromatography–tandem mass spectrometry (UPLC–MS/MS). Spectral matching with a false discovery rate threshold of <0.01 enabled annotation of 1703 specialized metabolites (Table S1), which were classified into eight major categories. Alkaloids constituted 24.43% of the total metabolites, with BIAs accounting for nearly half (49.04%) (Fig. 1A). Hierarchical clustering analysis revealed distinct metabolite accumulation patterns between samples, with those from rhizomes and fibrous roots forming two primary clusters that further subdivided into species-specific subclusters (Fig. 1B). Multivariate statistical analyses, including principal component analysis (PCA) and orthogonal partial least squares-discriminant analysis, corroborated this hierarchical organization (Fig. S1A and B). For instance, the first principal component (PC1), explaining 45.22% of total variance, clearly distinguished metabolites of rhizomes from those of fibrous roots, while the second component (PC2, 26.95%) captured interspecies variation (Fig. S1B). Cross-tissue correlation analysis also reinforced these findings, indicating that tissue type exerts a stronger influence than species differences in shaping metabolic profiles (Fig. S1C).

Comparative metabolomics revealed *C. chinensis* as a hyperaccumulator of diverse BIAs in both rhizomes and fibrous roots (Fig. 1C and D). Using a threshold of |log₂ (fold change)| > 1 and *P* < 0.05, we identified significantly elevated levels of BIAs such as epiberberine, jatrorrhizine, berberine, and palmatine in *C. chinensis* rhizomes compared to *C. teeta* (Fig. 1C). Kyoto Encyclopedia of Genes and Genomes (KEGG) enrichment analysis of these upregulated metabolites revealed strong associations with isoquinoline alkaloid biosynthesis, tropane/piperidine/pyridine alkaloid biosynthesis, and general alkaloid biosynthesis pathways (Fig. 1D). A similar trend was observed in fibrous roots, where *C. chinensis* accumulated substantially higher content of (*S*)-canadine, 3′-hydroxy-*N*-methylcoclaurine, jatrorrhizine, columbamine, coclaurine, berberine, and epiberberine than *C. teeta* (Fig. 1C). These differentially accumulated metabolites were similarly enriched in alkaloid biosynthesis pathways, including isoquinoline alkaloid biosynthesis, indole alkaloid biosynthesis, and the biosynthesis of various other alkaloids (Fig. 1D). These findings collectively establish *C. chinensis* as a superior species for BIA production (particularly epiberberine, palmatine and jatrorrhizine), with rhizomes serving as the principal biosynthetic tissue (Fig. S2).

### Species-divergent BIA compartmentalization and abundance during biosynthesis

**Figure 2:**
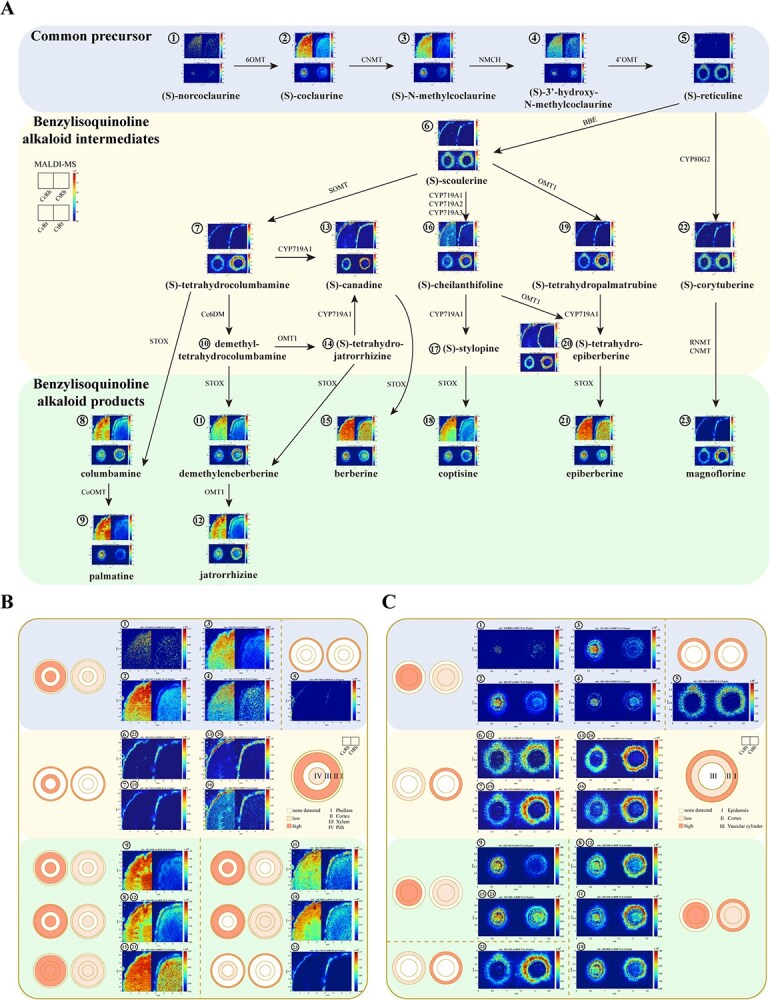
Species-specific compartmental distribution of BIAs in rhizomes and fibrous roots of *Coptis* determined using MALDI-MSI. (A) Spatial localization of 20 key metabolites in the BIA biosynthetic pathway across rhizomes and fibrous roots of *C. chinensis* and *C. teeta.* (B) Comparative analysis of compartmental distribution patterns of BIA-related metabolites in rhizomes of *C. chinensis* and *C. teeta*. (C) Comparative analysis of compartmental distribution patterns of BIA-related metabolites in fibrous roots of *C. chinensis* and *C. teeta*. Each image was obtained by selecting the exact mass of the protonated BIA-related metabolites (Table S2) and normalizing by the total ion current. The color scale represents signal intensity.

To elucidate the spatial localization of BIA pathway metabolites across different tissue compartments of the *Coptis* rhizomes and fibrous roots, we employed MALDI-MSI analysis to visualize the *in situ* distribution of 20 key metabolites in both *C. chinensis* and *C. teeta*. The analyzed compounds comprised five common precursors, seven BIA intermediates, and eight BIA products along the biosynthetic pathways (Fig. 2A, Table S2). The resulting imaging analysis revealed compartmentalized accumulation patterns of BIAs within specific tissue regions. In rhizomes, these specialized metabolites exhibited differential accumulation in four concentric tissue layers: phellem, cortex, xylem, and pith. Comparatively, in fibrous roots, the metabolites displayed distinct partitioning across three anatomical compartments: exodermis, cortex, and vascular cylinder (Fig. 2B and C).

The accumulation patterns and abundances of these BIAs within the same tissues differed significantly between *C. chinensis* and *C. teeta*, with compartmental distribution profiles varying according to metabolite identity (Fig. 2B and C). For instance, in rhizome tissues, the five common precursors exhibited conserved compartmental distribution patterns in both species, but their abundances were different, in which (*S*)-norcoclaurine, (*S*)-coclaurine, (*S*)-*N*-methylcoclaurine, and (*S*)-3′-hydroxy-*N*-methylcoclaurine were enriched in the cortical and pith, with significantly higher abundance in *C. chinensis* than in *C. teeta*. In contrast, the seven BIA intermediates displayed pronounced interspecies divergence in both compartmental distribution and abundance. While these intermediates preferentially accumulated in both the phellem and xylem in *C. chinensis*, they were largely restricted to the phellem in *C. teeta*. Among the BIA end products, palmatine (cortex/pith), columbamine/jatrorrhizine (phellem/cortex/pith), and berberine/epiberberine (cortex/xylem/pith) shared identical tissue distribution patterns between both species but were more abundant in *C. chinensis*. Conversely, demethyleneberberine (*C. chinensis*: phellem/cortex/pith; *C. teeta*: phellem/cortex), coptisine (*C. chinensis*: phellem/cortex; *C. teeta*: pan-tissue), and magnoflorine (*C. chinensis*: phellem/xylem; *C. teeta*: phellem) exhibited species-specific localization. Notably, demethyleneberberine and coptisine showed high abundance in *C. chinensis*, whereas magnoflorine levels remained comparable between the two species (Fig. 2B).

In fibrous roots, BIA metabolites similarly exhibited species-specific compartmental distribution patterns and abundance variations (Fig. 2C). While the common precursors maintained conserved localization profiles, the accumulation levels of (*S*)-norcoclaurine, (*S*)-coclaurine, (*S*)-*N*-methylcoclaurine, and (*S*)-3′-hydroxy-*N*-methylcoclaurine were significantly higher in *C. chinensis* than in *C. teeta*. All detected BIA intermediates exclusively accumulated in the epidermis but exhibited significantly higher abundance in *C. teeta*. Among the BIA end products, palmatine, berberine, and epiberberine were localized in the cortex and vascular cylinder in both species, though with greater abundance in *C. chinensis*; magnoflorine showed epidermis-specific accumulation and was markedly more abundant in *C. teeta*; columbamine, demethyleneberberine, jatrorrhizine, and coptisine displayed distinct species-dependent patterns, primarily accumulating in the cortex and vascular cylinder in *C. chinensis*, but were pan-tissue distributed in *C. teeta*, with relatively strong epidermal enrichment (Fig. 2C).

Collectively, the MALDI-MSI analyses revealed two fundamental trends: surface-tissue specific accumulation of (*S*)-reticuline (common precursor stage), all BIA intermediates, and magnoflorine (BIA product stage) versus internal-tissue preferential distribution of the remaining 11 metabolites, with particularly strong cortex-specific hyperaccumulation in *C. chinensis* rhizomes (Fig. 2B and C).

### Anatomical divergence underlies higher BIA accumulation in *C. chinensis* rhizomes

**Figure 3:**
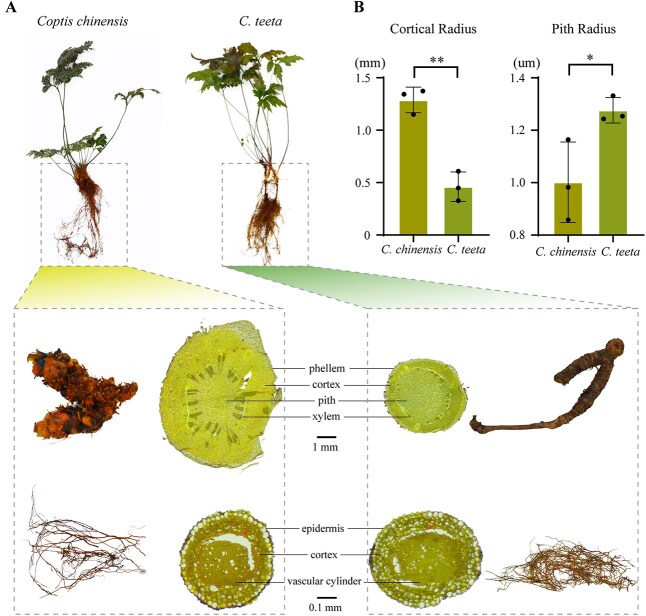
Morphological and anatomical comparison of *C. chinensis* and *C. teeta*. (A) Whole-plant morphology of *C. chinensis* and *C. teeta*, with magnified views of rhizomes (upper regions) and fibrous roots (lower regions), alongside their transverse section optical microscopic characteristics. Position indicated by arrow corresponds to the endodermis in fibrous root cross-section. (B) Comparative analysis of cortical radius and pith radius between *C. chinensis* and *C. teeta* (Student’s *t*-test; ^*^P < 0.05; ^**^P < 0.01).

The biosynthesis, transport, and storage of specialized metabolites are fundamentally governed by morphological and anatomical features of plant tissues [18]. To investigate the mechanisms underlying interspecies variation in BIA accumulation, we conducted a comparative analysis of the structural characteristics of rhizomes and fibrous roots in *C. chinensis* and *C. teeta*. Morphological examination revealed striking interspecies differences; *C. chinensis* rhizomes exhibited a stout, abbreviated morphology with characteristic chicken claw-like curvature and nodular protrusions on their surface (Fig. 3A). In contrast, *C. teeta* rhizomes displayed elongated, whip-like architecture with either smooth surfaces or subtle longitudinal striations (Fig. 3A). Their fibrous roots displayed equally distinct morphological specialization; *C. chinensis* produced sparse, short roots with swollen tips, while *C. teeta* developed dense and elongated fibrous roots (Fig. 3A).

Histological analysis also revealed anatomical differences of *C. chinensis* and *C. teeta* in both rhizomes and fibrous roots (Fig. 3 and Fig. S1). While both species exhibited well-defined rhizome stratification (phellem, cortex, xylem, pith), *C. chinensis* showed bright yellow pigmentation in cortical and pith parenchyma cells versus yellowish-green pigmentation in *C. teeta* (Fig. 3A). This is consistent with their divergent rhizome developmental allocation; *C. chinensis* possessed cortical expansion (1288.6 ± 121.6 μm) with pith reduction (1011.6 ± 153.9 μm), whereas *C. teeta* exhibited the inverse organization (Fig. 3B). The species further diverged in xylem structure, with *C. chinensis* developing robust, radially arranged wood fibers compared to *C. teeta*’s less defined xylem. Similarly, both species exhibited differential staining and structural features in their fibrous roots despite sharing a conserved anatomical architecture: epidermis (comprising a single layer of translucent to pale yellow cells), cortex (formed by multiple layers of loosely arranged parenchyma cells), and vascular cylinder (Fig. 3A). However, distinct pigmentation patterns were observed; *C. chinensis* displayed uniform yellow-brown pigmentation throughout its ground tissue, while *C. teeta* exhibited a characteristic dark green cellular organization (Fig. 3A). Furthermore, *C. chinensis* developed a well-defined endodermis that clearly demarcated the boundary between cortical and vascular tissues, which was absent in *C. teeta*.

These interspecies anatomical divergences, particularly in pigmentation patterns, cortical development, xylem lignification, and endodermal differentiation (Fig. 3), likely constitute evolutionary adaptations enhancing specialized metabolite biosynthesis and transport. Notably, 11 metabolites, including seven BIA end products and four metabolites from common precursor synthesis phase, exhibited pronounced cortical enrichment in *C. chinensis* rhizomes (Fig. 2A and B). The well-developed cortex in *C. chinensis* rhizomes provides substantial storage capacity for these metabolites, explaining their higher accumulation compared to *C. teeta* rhizomes (Fig. 3). Taken together, these findings establish that divergent cortical anatomy between *C. chinensis* and *C. teeta* constitutes the structural basis for interspecies differences in BIA accumulation.

### Elevated expression of BIA biosynthetic pathway genes mediates enhanced metabolite accumulation in *C. chinensis* rhizomes

To elucidate the molecular divergence between *C. chinensis* and *C. teeta*, we conducted comparative transcriptome analysis of rhizome and fibrous root tissues from both *Coptis* species. High-throughput sequencing generated millions of clean reads per tissue, identifying 35 217 expressed genes across all samples (Table S3). Hierarchical clustering, PCAs, and correlation matrix analysis revealed that transcriptomic divergence was predominantly driven by tissue type rather than interspecific variation (Fig. S3A–C), consistent with our widely targeted metabolomics data. Comparative expression profiling identified 618 significantly upregulated and 701 downregulated genes in *C. chinensis* rhizomes versus *C. teeta* (Figs S3D and S4). KEGG enrichment analysis showed isoquinoline alkaloid biosynthesis as one of the most significantly enriched pathways among these upregulated rhizome genes (Fig. S3E), corresponding to elevated alkaloid content in *C. chinensis* rhizomes (Fig. 1C and D). Similarly, upregulated genes in *C. chinensis* fibrous roots were significantly enriched in this pathway (Fig. S3F and G), indicating coordinated transcriptional and metabolic enhancement of BIA biosynthesis (Fig. 1C and D). Differential expression analysis combined with KEGG pathway enrichment identified eight key genes (*NCS5, CNMT2, BBE, CYP80G2, OMT1, CoOMT2, CoOMT4, CoOMT5*) in the BIA biosynthesis pathway (Fig. S5, Table S4), which likely contribute to the increased BIA accumulation in *C. chinensis*. Their higher expression in *C. chinensis* was validated by real-time quantitative polymerase chain reaction (RT-qPCR) (Fig. S6).

**Figure 4:**
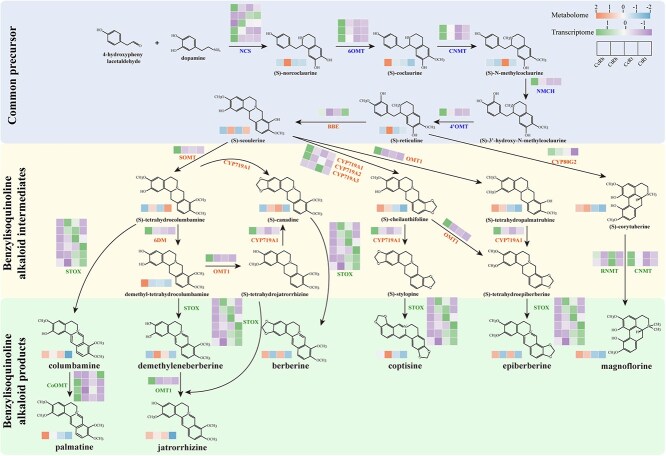
Integrated multi-omics visualization of the BIA biosynthesis pathway in *Coptis* species. Gene expression levels of BIA pathway genes are shown upon their corresponding enzymes, and metabolite contents are displayed beneath the structural formulae of the compounds. The biosynthetic pathway is organized into three sections: the early stage (common precursor synthesis), the middle stage (BIA intermediate synthesis), and the late stage (BIA product synthesis).

Integrated metabolomic and transcriptomic analyses further demonstrated strong concordance between datasets (Fig. 4). In the common precursor synthesis phase, five key pathway genes were identified, and most of the encoding genes (3/5 *CcNCS*, all *Cc6OMT* and *CcCNMT*, *CcNMCH* and *Cc4*′*OMT*) showed rhizome-specific upregulation in *C. chinensis*. This expression pattern correlated with MALDI-MSI-detected enrichment of early intermediates (*S*)-norcoclaurine through (*S*)-3′-hydroxy-*N*-methycoclaurine in *C. chinensis* rhizomes (Fig. 2A). The widely targeted metabolomics data also showed partial correlation, with (*S*)-coclaurine (Cc6OMT product) significantly accumulated in *C. chinensis* rhizomes (Fig. 4). Six pathway genes characterized the BIA intermediate phase, featuring elevated expression of *CcBBE* (second-highest), *CcSOMT*, *CcCYPs* (*CcCYP719A1*, *CcCYP719A2*, *CYP80G2*), *CcOMT1*, and *Cc6DM* in *C. chinensis* rhizomes, corresponding to tissue-specific accumulation of their products (Fig. 4). Furthermore, MALDI-MSI data partially validated this observation, revealing a notably abundant distribution of (*S*)-stylopine in the phellem and cortex tissues of *C. chinensis* rhizome (Fig. 2A). In the terminal phase, *CoOMT* exhibited markedly rhizome-specific expression in *C. chinensis*, with three out of the four associated genes showing significant upregulation (Fig. 4). This drove high palmatine accumulation in *C. chinensis* rhizomes as confirmed by both metabolomics and MALDI-MSI imaging (Figs 2A and 4). Overall, 11 of 14 BIA pathway genes exhibited coordinated upregulation in *C. chinensis* rhizomes, explaining its characteristic BIA accumulation pattern.

### Core TFs drive BIA biosynthesis and accumulation in *C. chinensis* rhizomes through direct gene activation

**Figure 5:**
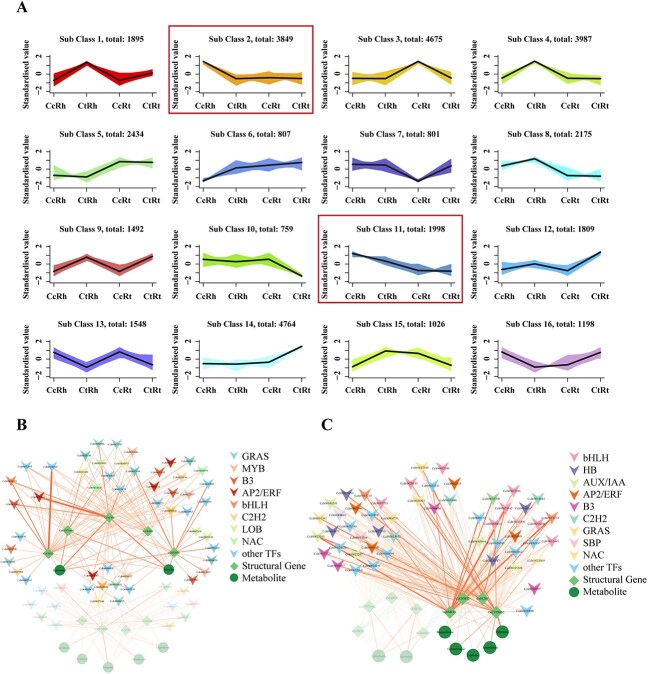
Dynamics of gene expression across the *C. chinensis* and *C. teeta* samples. (A) *k*-means clustering grouped the expression profiles into 16 subclasses. The *x*-axis depicts samples [*C. chinensis* rhizome (CcRh), *C. teeta* rhizome (CtRh), *C. chinensis* fibrous root (CcRt), and *C. teeta* fibrous root (CtRt)], and the *y*-axis depicts the Z-score standardized per gene. (B) Class 2 and (C) Class 11 co-expression correlation networks integrating TFs, structural genes, and metabolites. Triangles represent TFs (color-coded by family), diamonds denote structural genes, and circles indicate metabolites. The thickness of the lines is proportional to the correlation strength.

**Figure 6:**
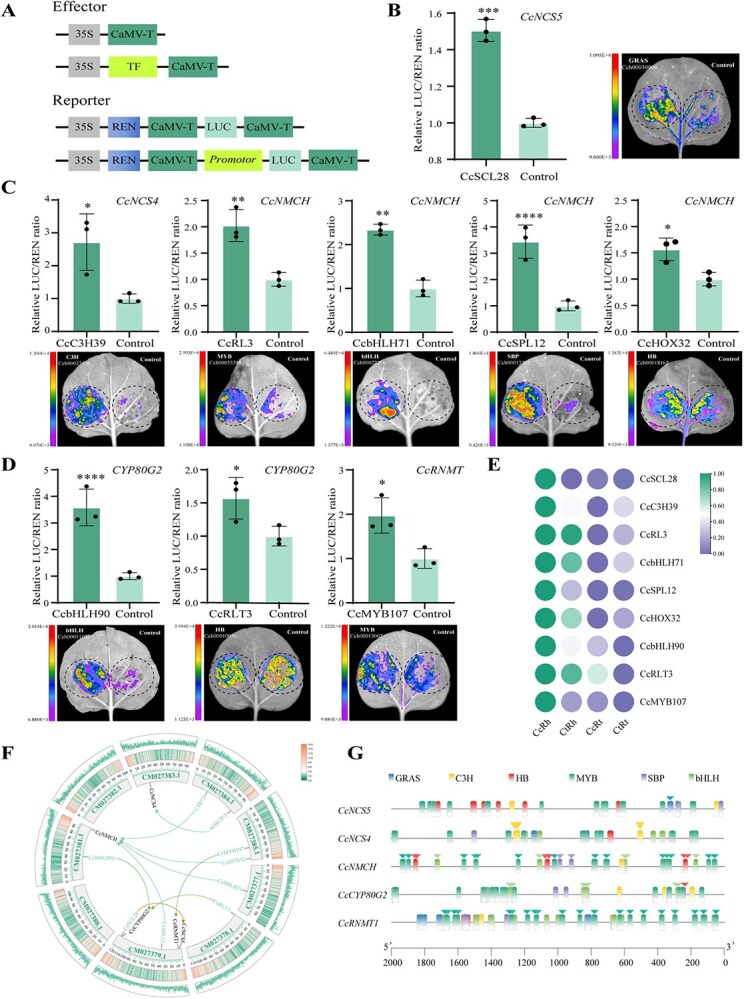
Dual-Luc assay validation. (A) Schematic diagram of Dual-Luc reporter vector construction. (B and C) Transcriptional activation validation of distinct regulatory modules: (B) CcSCL28 → *CcNCS5* → berberine/epiberberine; (C) CcC3H39/CcRL3/CcbHLH71/CcSPL12/CcHOX32 → *CcNCS4/CcNMCH* → various terminal BIAs; (D) CcbHLH90/CcRLT3/CcMYB107 → *CcCYP80G2/CcRNMT1* → magnoflorine (Student’s *t*-test; ^*^P < 0.05; ^**^P < 0.01; ^***^P < 0.001; ^****^P < 0.0001). (E) Expression heatmap of nine TFs across *Coptis* tissues. (F) Chromosomal mapping of key TFs and structural genes. (G) TF binding site analysis in promoters of five structural genes.

Given tissue-specific hyperexpression of enzymatic genes is coordinately regulated, we explored the regulatory mechanisms underlying BIA biosynthesis in *Coptis* through *k*-means clustering of transcriptomic data. This analysis categorized co-expressed genes into 16 distinct subclasses, of which two specific transcriptional subclasses (Classes 2 and 11) exhibited robust correlation with BIA metabolic flux across different tissues, with expression profiling that closely mirrored both quantitative metabolite data and MALDI-MSI spatial distribution patterns (Fig. 5A). These subclasses contained 20 structural genes (14 in Class 2, 6 in Class 11) essential for BIA biosynthesis (Fig. S7). Co-expression network analysis revealed seven BIA metabolites strongly correlated with nine structural genes, delineating two upstream regulatory modules in *C. chinensis*; Class 2 governed the biosynthesis of berberine and epiberberine by coordinating the upstream genes *CcNCS5*, *CcCYP719A2*, and *CoOMT2/4* (Fig. 5B), while Class 11 mediated the production of jatrorrhizine, coptisine, palmatine, and magnoflorine through the coordinated expression of *CcNCS4*, *CcCNMT2*, *CcNMCH*, and *CcCYP80G2* (Fig. 5C). Guided by co-expression network, 36 high-confidence TF-structural gene regulatory pairs were predicted (Table S5), and 26 successfully cloned TFs (spanning 27 TF-structural gene combinations) were further experimentally validated for their interactions through dual-luciferase (Dual-Luc) reporter assays. As a result, nine TFs from six families that transcriptionally regulate the BIA biosynthetic pathway in *C. chinensis* were identified (Fig. 6 and Fig. S8). These regulatory functions originate from the two co-expression modules of Class 2 (Fig. 6B) and Class 11 (Fig. 6C and D) mentioned previously.

The Class 2 regulatory network is governed by CcSCL28 (Cch00030906), a GRAS-family TF homologous to *Arabidopsis thaliana* SCARECROW-LIKE28, which regulates cell and organ development (Fig. 6B). CcSCL28 significantly enhanced *CcNCS5* transcription by 1.45-fold, establishing the GRAS → *CcNCS5* →berberine/epiberberine regulatory axis that indirectly promotes terminal BIA biosynthesis in *C. chinensis* (Fig. 6B). In contrast, the more complex Class 11 network involves MYB, bHLH, HB, C3H, and SBP family TFs regulating the biosynthesis of multiple BIAs (Fig. 6C). This network can be subdivided into two regulatory units based on their functional stages (Fig. 6C and D). The common precursor regulatory unit, comprising five TFs [CcC3H39 (Cch00023130, *A. thaliana* AtC3H39 homolog), CcRL3 (Cch00033398, *Oryza sativa* OsRL3 homolog), CcbHLH71 (Cch00022206, *Capsicum annuum* bHLH71 homolog), CcSPL12 (Cch00013307, *O. sativa* OsSPL12 homolog), CcHOX32 (Cch00018167, *Musa acuminata* HOX32 homolog)], orchestrates the common precursor stage of BIA biosynthesis. In *C. chinensis*, CcC3H39 activated *CcNCS4* (2.71-fold), while the other four TFs activated *CcNMCH* (1.56- to 3.43-fold) (Fig. 6C), forming the CcC3H39/CcRL3/CcbHLH71/CcSPL12/CcHOX32 → *CcNCS4*/*CcNMCH* → end BIA products module. The magnoflorine terminal regulatory unit, consisting of three TFs [CcbHLH90 (Cch00011077, *Juglans regia* bHLH90 homolog), CcRLT3 (Cch00010956, *A. thaliana* AtRLT3 homolog), CcMYB107 (Cch00013067, *A. thaliana* AtMYB107 homolog)], directly regulates the final two steps of magnoflorine synthesis. CcbHLH90 and CcRLT3 enhanced *CcCYP80G2* expression (3.57- and 1.56-fold, respectively), while CcMYB107 activated *CcRNMT1* (1.96-fold), forming the CcbHLH90/CcRLT3/CcMYB107 → *CcCYP80G2*/*CcRNMT1* → magnoflorine molecular module (Fig. 6D). Collectively, these rhizome-specific TFs orchestrate highly compartmentalized BIA accumulation in *C. chinensis* through potentially direct transcriptional control of core pathway genes.

## Discussion

### Comparative accumulation of BIAs between *C. chinensis* and *C. teeta*

As medicinally important species within the Ranunculaceae family, *C. chinensis* and *C. teeta* represent crucial botanical resources used in TCM, possessing comparable BIA profiles. Existing research has established that *C. chinensis* accumulates higher contents of columbamine, coptisine, palmatine, epiberberine, and total alkaloids compared to other *Coptis* species, whereas *C. teeta* preferentially produces berberine, jatrorrhizine, and magnoflorine [14–16]. Our results not only confirmed these established biosynthetic trends but further revealed compound-specific quantitative variations: *C. chinensis* showed rhizome-dominant accumulation of berberine, epiberberine, columbamine, palmatine, and jatrorrhizine, while *C. teeta* exhibited elevated levels of demethyleneberberine and coptisine (Fig. 4). Notably, magnoflorine maintained comparable interspecific accumulation levels, exhibiting consistent rhizome-specific localization patterns in both (Fig. 4). Compared to prior reports, our results revealed elevated levels of both berberine and jatrorrhizine in *C. chinensis*, while a preferential accumulation of coptisine in *C. teeta*. The production of specialized metabolites in medicinal plants is known to exhibit substantial plasticity, influenced not only by genetic determinants but also by ecological and cultivation factors including geographical origin, harvest timing, and developmental stage [1, 6]. The observed differences between our results and earlier studies may therefore reflect variations in such sampling parameters, particularly growth years of *Coptis* plants, seasonal collection period, and cultivation conditions. Such influences of sampling conditions on interspecific variation in alkaloid profiles, particularly berberine accumulation, have been documented in a previous study, supporting the plausibility of this interpretation [15]. Based on comprehensive metabolomic analyses, we evidenced the superior medicinal quality of *C. chinensis* at the bioactive constituent level through its markedly elevated total alkaloid content and distinct epiberberine accumulation dynamics (Fig. 1C and D). These findings were also rigorously validated by spatially resolved metabolomic profiling and tissue-specific transcriptional regulation analyses, reflecting species-specific metabolite partitioning patterns, which potentially contribute to variable therapeutic efficacy among *Coptis* species.

### Structural specialization facilitates cortical storage of BIAs 

The accumulation of bioactive constituents in medicinal plants often exhibits strong tissue specificity, including the compartmentalized distribution patterns of secondary metabolites within specific tissues [37]. Recent technological advances in MALDI-MSI have enhanced our ability to investigate the spatial distribution of phytochemicals in medicinal plants [38]. Our MALDI-MSI analysis uncovered clear alkaloid compartmentalization in *Coptis* species, with a strong correlation between species-specific spatial partitioning of alkaloids and rhizome-fibrous root architectural specialization in *C. chinensis* versus *C. teeta* (Fig. 2). Notably, the significantly expanded cortical region in *C. chinensis* rhizomes appears to create specialized microdomains for alkaloid storage (Figs 2 and 3). Through comprehensive MALDI-MSI, we identified 11 metabolites exhibiting pronounced cortical enrichment in *C. chinensis* rhizomes, consistent with the enhanced cortical development (Figs 2 and 3). This spatial differentiation of specialized metabolites mirrors common compartmentalization patterns in medicinal plants. For instance, the cortex of the Nail head structure in *Panax notoginseng* is more developed than in the main root, and MALDI-MSI localized ginsenoside Rb1 predominantly within this cortical region [39]. This anatomical specialization corresponds to significantly higher ginsenoside Rb1 accumulation in the Nail head of *P. notoginseng*. The well-developed cortical structures in both the Nail head of *P. notoginseng* and the rhizomes of *C. chinensis* provide a structural foundation for the accumulation of their active compounds. These anatomical adaptations underscore the evolutionary optimization of rhizome architecture for enhanced secondary metabolite production, providing a structural basis for understanding the superior medicinal properties of *C. chinensis*.

### Multilayered enhancement of the BIA biosynthetic pathway 

Understanding tissue-specific biosynthesis of secondary metabolites in medicinal plants through pathway-specific associations provides critical insights into the molecular mechanisms governing their spatial accumulation patterns [40]. While BIA biosynthesis has been extensively studied in Papaveraceae species [41–45], research progress in Ranunculaceae remains limited [25, 27, 30, 35, 46–48]. Our study demonstrates multilayered transcriptional enhancement in the tyrosine-derived BIA pathway of *C. chinensis* rhizomes: precursor-phase genes (3/5 *CcNCS*, all *Cc6OMT*, *CcCNMT*, *CcNMCH*, *Cc4*′*OMT*) show tissue-specific upregulation; intermediate-phase genes (*CcBBE*, *CcSOMT*, *CcCYP719A1/A2*, *CcCYP80G2*, *CcOMT1*, *Cc6DM*) exhibit significantly elevated expression; and terminal-phase multi-copy *CoOMT* genes display specific activation (Fig. 4). At the transcriptional level, coordinated upregulation of 11/14 key BIA biosynthetic enzymes accounts for the substantial accumulation of seven major BIA end products (excluding demethyleneberberine), and aligns with the rhizome-predominant accumulation profile (Figs 1, 2, and  4). The tight correlation between enhanced BIA accumulation and coordinated upregulation of biosynthetic genes in *C. chinensis* rhizomes provides molecular evidence explaining its different medicinal quality relative to *C. teeta*.

### Modular transcriptional regulation of BIA biosynthesis

TFs orchestrate plant specialized metabolism by regulating rate-limiting enzyme genes in biosynthetic pathways [49, 50]. Our study deciphers the BIA regulatory network in *C. chinensis* through *k*-means clustering and Dual-Luc reporter assays, identifying two distinct regulatory modules: a Class 2 module of GRAS → *CcNCS5* → berberine/epiberberine regulatory axis mediated by CcSCL28 (Fig. 6B) and a more complex Class 11 module (Fig. 6C and D). The latter includes five TFs (CcC3H39, CcRL3, CcbHLH71, CcSPL12, CcHOX32) that cooperatively activate common precursor synthesis genes (*CcNCS4* and *CcNMCH*), and three TFs (CcbHLH90, CcRLT3, CcMYB107) that directly drive the final steps leading to magnoflorine. This modular ‘precursor synthesis-terminal specialization’ strategy likely optimizes metabolic flux allocation and minimizes pathway interference. Previous studies have established the critical regulatory role of bHLH TFs in the BIA pathway of *Coptis* [33, 35]. For instance, members of bHLH subgroup Ib (CcbHLH001, CcbHLH002) primarily regulate structural genes like *CcBBE* and *CcCYP719*, which function during the intermediate biosynthetic stage of the BIA pathway [33]. In this study, we identified two novel bHLH genes, CcbHLH71 (subgroup IIIa) and CcbHLH90 (subgroup Ia). Functional characterization revealed that CcbHLH71 specifically activates the precursor synthesis gene *CcNMCH*, whereas CcbHLH90 activates the intermediate biosynthesis gene *CcCYP80G2* (Fig. S9). This suggests that bHLH TFs belonging to subfamily I likely share more similar functions in regulating the BIA biosynthetic pathway, activating structural genes during the intermediate biosynthetic stage. Our results thus demonstrated that distinct bHLH subgroups target specific stages and genes within BIA biosynthesis, highlighting their functional diversification.

## Conclusion

In summary, we propose an integrated mechanism explaining the differential BIA accumulation in *C. chinensis* relative to *C. teeta* through anatomical specialization and transcriptional regulation. The expanded rhizome cortex and fibrous root endodermis in *C. chinensis* provide enhanced storage capacity, while rhizome-specific TFs coordinately regulate BIA biosynthetic networks. This framework elucidates the mechanistic basis for tissue-specific phytochemical distribution in medicinal plants and advances our understanding of specialized metabolism regulation in nonmodel plant species.

## Materials and methods

### Plant materials

We collected 4-year-old cultivated *C. chinensis* and *C. teeta* plants in autumn (November 2022) from Lichuan, Hubei Province (30.30°N, 108.85°E), and Jinping, Yunnan Province (22.75°N, 103.25°E), China, respectively. The living plant materials have been preserved in the *Coptis* Resource Garden of Hubei University of Chinese Medicine, located in Lichuan, Hubei Province, with the sample codes Cc_LC_2022001 (*C. chinensis*) and Ct_YN_2022002 (*C. teeta*). After an ultrasonic cleaning in deionized water, we dissected the whole plants under sterile conditions to isolate their rhizomes and fibrous roots. For each species, the nine individuals were then arranged into three biological replicates, each comprising a pool of three individuals. Subsequently, the tissues were split into two processing workflows. Triplicate aliquots were snap frozen in liquid nitrogen and stored at −80°C for transcriptomic and widely targeted metabolomic analyses. In parallel, we processed the other freshly dissected specimens for MALDI-MSI within 2 h to preserve their native spatial metabolite distributions.

### LC–MS/MS analysis of metabolites

We vacuum lyophilized tissue samples for 63 h at −50°C using a Scienta-100F freeze dryer, cryo-pulverized them in liquid nitrogen-cooled vessels (30 Hz, 90 s; Mixer Mill MM 400, Retsch), and sieved the resulting powder through a 250-μm mesh. We then extracted 50.0 ± 0.1 mg of the homogenized powder with 1.2 ml of ice-cold methanol/water (70:30, v/v; −20°C) containing an internal standard (2-chlorophenylalanine). After performing six cycles of vortex mixing (30 s) and centrifugation (12 000*g*, 4°C, 3 min), we filtered the supernatants through a 0.22-μm membrane for analysis. Metabolites were separated on an Agilent Poroshell 120 SB-C18 column (2.1 × 100 mm, 1.8 μm; 40°C) using a 14-min gradient of 0.1% (v/v) formic acid in water (A) and acetonitrile (B) at 0.35 ml/min: 5% B (0 min), linear increase to 95% B (9 min), 95% B (9–10 min), return to 5% B (11.1 min), and re-equilibration (11.1–14 min). We performed detection in scheduled multiple reaction monitoring (MRM) mode on a Sciex ExionLC™-AD UPLC-triple-quadrupole MS system. Metabolite identification was based on MS/MS spectral matching against the MetWare MWDB v2.0 database, excluding isotopes, adducts, and fragment ions. For quantification, we used optimized MRM transitions, which were validated by daily quality controls (retention time drift <5%; intensity variation <15%), and normalized peak areas across samples.

### MALDI-MSI and MS/MS imaging

We transversely cryo-sectioned snap-frozen tissues into 18-μm sections at −20°C using a Leica CM3050 cryostat with deionized water as an adhesive, vacuum dried the sections on slides for 10 minutes, and acquired topographic maps (Fig. S10). For MALDI-MSI, we applied the matrix using an automated sprayer (flow rate: 2400 μl/h; sheath gas: 0.35 MPa; nozzle temperature: 100°C; traverse speed: 0.17 m/s; voltage: 6 kV; distance: 12 cm). Full-scan MSI was performed on a 7 T solariX FTICR MS (Bruker) equipped with a Smartbeam II laser (repetition rate: 1 kHz; laser spot diameter: 30 μm; power: 35%; step size: 35 μm). The instrument was externally calibrated using DHB peaks and Peptide Calibration Standard II across the m/z ranges of 100–2100 (positive ion mode) and 100–1000 (negative ion mode). We processed the TIC-normalized images using Data Analysis 4.0 and FlexImaging 4.1 software. For MS/MS imaging, we employed an AP-SMALDI10 source (TransMIT) coupled to a Q-Exactive Orbitrap MS (Thermo Fisher) with a 343-nm laser (2000 Hz) in positive ion mode (isolation width: 0.9 Da; step size: 50 μm; resolution: 140 k at m/z 200). Data analysis for MS/MS imaging was conducted using MIRION software [51].

### Transcriptome sequencing and assembly

Total RNA was isolated from fibrous roots of *C. chinensis* and *C. teeta* using TRIzol® reagent (Thermo Fisher Scientific, Waltham, MA, USA). Strand-specific cDNA libraries were constructed and sequenced (150-bp paired-end) on an Illumina NovaSeq 6000 platform. After quality control filtering of the raw reads, we retained only high-quality sequences for subsequent analysis. We also retrieved publicly available rhizome transcriptome data for both species from the National Center for Biotechnology Information (NCBI) database (accession number: SRP132619). Using the combined datasets from rhizome and fibrous root transcriptomes, we performed a genome-guided transcriptome assembly against the *C. chinensis* reference genome with StringTie (v2.2.3) [31, 52]. Finally, we quantified gene expression levels as transcripts per million based on uniquely mapped reads using featureCounts (v2.0.2) [53].

### Differential analysis and KEGG pathway enrichment

We performed differential metabolite profiling in R (v3.5.1) with the MetaboAnalystR package (v1.0.1) using default parameters and applying significance thresholds of |log₂ (fold change)| > 1 and adjusted *P* < 0.05 [54]. Differential gene expression analysis was conducted with DESeq2 (v1.22.1) under identical statistical thresholds [55]. We generated volcano plots for both differentially expressed genes and metabolites using ggplot2 (v3.4.2) [56]. KEGG pathway enrichment analysis was implemented in Python (v3.6.6) utilizing the pandas library (v0.23.4) [57].

### Real-time quantitative PCR

Total RNA was isolated from rhizomes and fibrous roots of *C. chinensis* and *C. teeta* using the AFTMag Quick Complex Plant RNA Extraction Kit (ABclonal, RK30181), with concentration and purity quantified by NanoDrop spectrophotometry (Thermo Fisher Scientific) and integrity verified through 1.0% agarose gel electrophoresis (Bio-Rad PowerPac Basic System, Hercules, CA, USA). The extracted RNA was reverse transcribed into cDNA using ABScript Neo RT Master Mix with gDNA remover (ABclonal, RK20433), followed by RT-qPCR amplification with BrightCycle Universal SYBR Green qPCR Mix containing UDG (ABclonal, RK21219) on a Thermo Fisher QuantStudio 5 system (Thermo Fisher Scientific). We calculated gene expression levels using the 2^-ΔΔ^Ct method and determined statistical significance between species/tissues by Student’s *t*-tests in GraphPad Prism (v9.0) (GraphPad Software, San Diego, CA, USA). Primer sequences used for RT-qPCR are provided in Table S6. To generate the standard curve, a fixed concentration of cDNA was serially diluted 10-fold to produce six concentration gradients (C1 to C6). Each dilution was subjected to RT-qPCR amplification with eight distinct primer pairs, with all reactions performed in triplicate. The standard curve was constructed by plotting the logarithmic template concentration (Log₁₀[C]) against the measured Ct values (Fig. S11). Subsequently, the amplification efficiency (*E*) for each primer was derived from the curve’s slope using the formula *E* = [10^(−1/slope)^ – 1] × 100% (Fig. S11).

### *k*-means clustering analysis and co-expression network construction

Transcriptome data were subjected to *k*-means clustering using the stats package (v4.2.0) in R (v4.2.1) [58]. We calculated Pearson correlations between BIA end product content and structural gene expression levels, and identified significant correlations at *r* > 0.7 and *P* < 0.05. Target gene clusters of interest were selected from *k*-means groupings, followed by correlation analysis between TFs within these clusters and the filtered structural genes. We constructed an integrated TF-structural gene-metabolite co-expression network and visualized it in Cytoscape (v3.9.1) using a force-directed layout algorithm, with edge weights proportional to correlation coefficients [59].

### Dual-Luc assays

We cloned the coding sequences of candidate TFs into the pGreen II 62-SK effector vector by homologous recombination and inserted the promoter regions (2000-bp upstream of each structural gene) into the pGreen II 0800-SK reporter vector. Both effector and reporter were co-transformed into the *Nicotiana benthamiana* leaves by using *Agrobacterium tumefaciens* strain GV3101(pSoup). Using a needleless syringe, we infiltrated bacterial suspensions into the abaxial epidermis of *N. benthamiana* leaves and then incubated the plants for 72 h under 16/8-h light/dark conditions at 22°C. Luminescence signals were quantified using the Dual-Luciferase Reporter Assay System (Promega, E1910) according to the manufacturer’s protocol. The transcriptional regulation ability of candidate TFs was calculated as the LUC/REN ratio normalized to empty vector controls. Primer sequences used for Dual-Luc assays are provided in Table S7.

### Bioinformatics analysis

We performed chromosomal localization and visualization using TBtools II (*Gene Density Profile* and *Gene Location Visualize from GTF/GFF* modules) [60]. TF binding sites were predicted via Plant Transcriptional Regulatory Map’s *Binding Site Prediction* module (https://plantregmap.gao-lab.org/) [61], with visualization implemented using TBtools’ *Gene Structure View* function [60]. We downloaded the selected bHLH TF protein sequences from the NCBI database. Multiple sequence alignment was performed using the MUSCLE algorithm with default parameters in MEGA (v11.0.13) [62]. Phylogenetic reconstruction was then conducted via the neighbor-joining (NJ) method with 1000 bootstrap replicates under the Jones–Taylor–Thornton substitution model.

### Statistical analysis

All experiments were performed with a minimum of three independent biological replicates. Data are presented as mean ±standard deviation (SD). Statistical significance was determined by Student’s *t*-tests with the following thresholds: ^*^P < 0.05, ^**^P < 0.01, ^***^P < 0.001, and ^****^P < 0.0001.

## Supplementary Material

Web_Material_uhaf338

## Data Availability

All data supporting the findings of this study are available within the paper and its supplementary data published online. Raw RNA-seq reads generated in this study have been deposited in the National Genomics Data Centre (https://ngdc.cncb.ac.cn/) under project no. PRJCA044774.
