# Supplementary material for: Multi-omics analysis reveals structural and transcriptional regulation specificity underlying differential benzylisoquinoline alkaloid accumulation in Coptis
Source: Hortic Res. 2025 Dec 15;13(3):uhaf338. doi: 10.1093/hr/uhaf338 (PMC12977165; doi:10.1093/hr/uhaf338)
Supplement: Web_Material_uhaf338 [file web_material_uhaf338.zip › Supplementary Figures-R.doc]

**Supplementary Figures**

**Fig. S1** Metabolomics analysis of *C. chinensis* and *C. teeta* rhizomes and fibrous roots.

**Fig. S2** Analysis of differential metabolites between the rhizome and fibrous roots of *C. chinensis.*

**Fig. S3** Comparative transcriptomic analysis between *C. chinensis* and *C. teeta*.

**Fig. S4** Upset plot of differentially expressed genes across various comparison combinations.

**Fig. S5** Venn diagram of differentially expressed genes and pathway genes involved in isoquinoline alkaloid biosynthesis in *C. chinensis*.

**Fig. S6** Relative expression levels of differentially expressed structural genes in the rhizomes and fibrous roots of *C. chinensis* and *C. teeta.*

**Fig. S7** Venn diagram of key *k*-means classes and pathway genes associated with isoquinoline alkaloid biosynthesis in *C. chinensis.*

**Fig. S8** Dual-luciferase assay results of eighteen combinations that did not show significant transcriptional activation.

**Fig. S9** Phylogenetic tree of select bHLH transcription factors.

**Fig. S10** Sample Schematic of MALDI-MSI.

**Fig. S11** Standard curves and amplification efficiencies for the eight RT-qPCR primer pairs.

**Fig. S1** Metabolomics analysis of *C. chinensis* and *C. teeta* rhizomes and fibrous roots. **(A)** OPLS-DA plot. **(B)** PCA plot. **(C)** Correlation analysis of metabolites in rhizomes and fibrous roots of *C. chinensis* and *C. teeta.*

**Fig. S2** Analysis of differential metabolites between the rhizome and fibrous roots of *C. chinensis.* **(A)** Volcano plots of differential metabolites between rhizomes and fibrous roots of *C. chinensis.* **(B)** KEGG enrichment analysis of upregulated differential metabolites in rhizomes compared to fibrous roots of *C. chinensis*.

**Fig. S3** Comparative transcriptomic analysis between *C. chinensis* and *C. teeta*. **(A)** Heat map of total expressed genes in *C. chinensis* rhizome (CcRh), *C. teeta* rhizome (CtRh), *C. chinensis* fibrous root (CcRt), and *C. teeta* fibrous root (CtRt). **(B)** PCA analysis of transcriptome data of *C. chinensis* and *C. teeta*. **(C)** The hierarchical clustering analysis of expression profiles of 35,217 genes from *C. chinensis* and *C. teeta* samples. **(D)** Volcano plot illustrating differential expressed genes between *C. chinensis* rhizome and *C. teeta* rhizome. **(E)** KEGG enrichment analysis of upregulated differential expressed genes in *C. chinensis* rhizome compared to *C. teeta* rhizome. **(F)** Volcano plot illustrating differential expressed genes between *C. chinensis* fibrous root and *C. teeta* fibrous root. **(G)** KEGG enrichment analysis of upregulated differential expressed genes in *C. chinensis* fibrous root compared to *C. teeta* fibrous root.

**Fig. S4** Upset plot of differentially expressed genes across various comparison combinations. Green dots in the plot denote gene sets that are found only in specific combinations or in a few combinations connected by lines. The bar chart on the left shows the number of genes in each combination, while the bar chart at the top displays the number of genes in the gene sets represented by the green dots.

**Fig. S5** Venn diagram of differentially expressed genes and pathway genes involved in isoquinoline alkaloid biosynthesis in *C. chinensis.*

**Fig. S6** Relative expression levels of differentially expressed structural genes in the rhizomes and fibrous roots of *C. chinensis* and *C. teeta.*

**Fig. S7** Venn diagram of key *k*-means classes and pathway genes associated with isoquinoline alkaloid biosynthesis in *C. chinensis*.

**Fig. S8** Dual-luciferase assay results of eighteen combinations that did not show significant transcriptional activation.

**Fig. S9** Phylogenetic tree of selected bHLH transcription factors.

**Fig. S10** Sample schematic of MALDI-MS imaging.

**Fig. S11** Standard curves and amplification efficiencies for the eight RT-qPCR primer pairs.
